# Real-World Synthetic Biology: Is It Founded on an Engineering Approach, and Should It Be?

**DOI:** 10.3390/life9010006

**Published:** 2019-01-07

**Authors:** Jamie A. Davies

**Affiliations:** UK Centre for Mammalian Synthetic Biology, University of Edinburgh, Edinburgh EH8 9YL, UK; jamie.davies@ed.ac.uk

**Keywords:** standardization, modularity, abstraction, modelling, design, assay

## Abstract

Authors often assert that a key feature of 21st-century synthetic biology is its use of an ‘engineering approach’; design using predictive models, modular architecture, construction using well-characterized standard parts, and rigorous testing using standard metrics. This article examines whether this is, or even should be, the case. A brief survey of synthetic biology projects that have reached, or are near to, commercial application outside laboratories shows that they showed very few of these attributes. Instead, they featured much trial and error, and the use of specialized, custom components and assays. What is more, consideration of the special features of living systems suggest that a conventional engineering approach will often not be helpful. The article concludes that the engineering approach may be useful in some projects, but it should not be used to define or constrain synthetic biological endeavour, and that in fact the conventional engineering has more to gain by expanding and embracing more biological ways of working.

## 1. Introduction

Most contemporary synthetic biology [1], as distinct from the first-era synthetic biology of the late 19th and early 20th century [2], aims to build new properties into living systems so that they do or make something useful. Common goals include creation of organisms that synthesize therapeutic or industrial molecules, that produce biofuels efficiently from waste or solar energy, that remediate contaminated land, that detect and report environmentally or medically significant molecular markers, or that manipulate or report on the activities of natural organisms for scientific investigation (reviewed by [3,4,5,6]). There is, in addition, a quite different aspect of synthetic biology, which aims to create life from non-living constituents: the character and aims of this type of synthetic biology are so different from the biotechnological aspects mentioned above that it will not be discussed further here. Reviews of its history and current status are available elsewhere [1,7].

The word ‘engineering’ permeates most introductions to synthetic biology, and it is used in two distinct ways. The first use, as a simple verb, refers merely to the physical process of altering the cell, as in the phrase ‘engineering a cell to make substance X’; this use is trivial and will not be discussed further. The second use of ‘engineering’, as a noun, evokes the outlook, working practices and technical culture of the engineering profession. This use appears because a very large number of influential accounts of 21st-century synthetic biology claim either that synthetic biology should adopt the engineering approach [8] or that it already does [9,10,11,12]. Indeed, an engineering outlook has been used by some authors as their way of distinguishing synthetic biology from other types of biotechnology. Examples of these statements include; “Synthetic biology is a recently emerging field that applies engineering formalisms to design and construct new biological parts, devices, and systems for novel functions or life forms that do not exist in nature.” [9]; “A key aspect of synthetic biology… is the application to biology of techniques which are normally used in engineering design and development. … Systems are normally built from standard devices, which in turn are built from standard parts. The standard parts and devices are all fully characterised and may be used in the design of multiple systems...” [10]; “ Synthetic biology…. combines the investigative nature of biology with engineering design principles” [11]; “Building novel parts, devices and in particular complex systems will require a systematic approach that relies on modularity and abstraction... This is synonymous to following an engineering approach. … Synthetic biology with its engineering vision aims to overcome the existing fundamental inabilities in system design and system fabrication...” [12].

The engineering approach outlined in the above quotations, and explained in more detail in the articles from which they come, differs from other methods of creation (e.g., those of arts and crafts) in several ways. Engineering usually involves a clear definition of the goal, followed by a careful design phase, which usually uses well-defined models that can predict, for example, the behaviour of a transistor when presented with a signal, or the bending of a bridge deck when presented with a load. Tasks and physical entities are usually divided into modules, and modules themselves are built from standard components (machine screws, resistors etc.), manufactured in bulk to reliable specifications. The behaviours of the components and the entities into which they are combined are measured and described in standard ways, and their interactions are well understood. Design is usually hierarchical; while an integrated circuit designer may worry about the details of his silicon chip, a computer motherboard designer can take the behaviour of each chip for granted and concentrate only on the interconnections of chips of different kinds to perform a high-level function, and a network designer can take the function of each computer in the network for granted and concentrate on the topology and performance of the network as a whole. A software engineer can take all of this for granted, and write her code for the applications that will run on these systems; typically, she will make use of libraries of existing code modules that are well characterised and can be re-used in a multitude of projects.

Engineering also involves a professional culture. Its practitioners generally subscribe to notions of professional knowledge, training, qualification and behaviour that effectively make them, too, into standardized, predictable components that will produce a broadly predictable and consistent outcome when applied to a well-defined task. This cultural aspect of engineering is seldom mentioned explicitly in articles about the importance of engineering approaches in synthetic biology, but it is implicit, for it would be very difficult for all other aspects of engineering to be adopted wholeheartedly without the adoption of the underlying culture.

In this review, I will begin by examining some successful synthetic biology projects of the last two decades, with a view to answering the question “is synthetic biology operating like a normal engineering discipline now?”. I will then go on to consider the pillars of conventional engineering culture in the light of synthetic biology, to address the question “Should it?”. The answers will be “No”, and “Not necessarily”.

## 2. Features of the Engineering Approach

Before asking whether successful synthetic biological projects of the 21st century really have followed an approach typical of conventional engineering, it is necessary to define what that approach is. Many authors have outlined the features of the engineering approach (e.g., [1,13,14,15,16,17,18,19]) and, while they do not agree on absolutely everything, their writings reach a broad consensus on the presence of the following features;

1. a design phase that uses predictive models
2. designing as much as possible using standard, well-characterised components
3. hierarchical design using functional modules
4. manufacture using reliable, quality-assured systems
5. testing of (at least samples of) finished devices using standardised measurement techniques.

**Figure 1:**
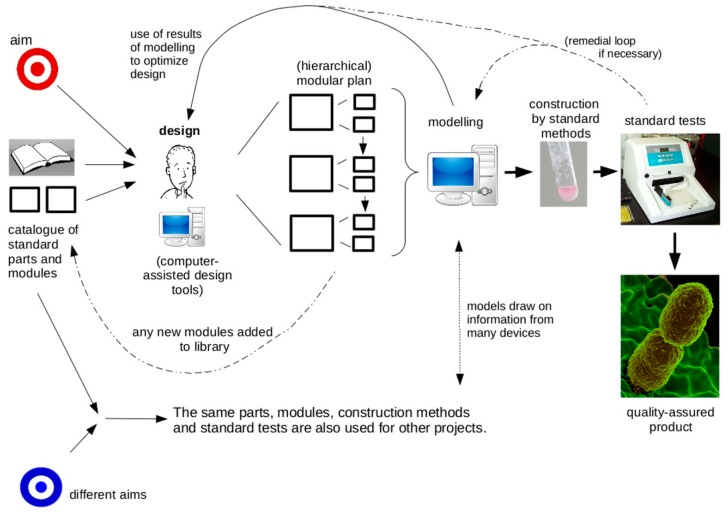
A diagrammatic representation of the ‘engineering approach’ discussed here. The design phase uses, as much as it can, standard components and pre-existing modules, themselves, made of standard components, to find a plan for the desired device. The plan will as far as possible divide the device into modules, which can be designed in detail separately (perhaps by separate teams). In complex projects, this modularity may be hierarchical, modules themselves being made of sub-modules etc. The design phase involves modelling, drawing on the known properties of the components and modules used, and on representations of the connections between them. This modelling is used to optimize the design before it is realized in wet-ware. Actual construction and testing are by standard methods, and in the unlikely event that the device does not work as intended, its actual behaviour is fed back to modelling to improve the design. The result is a quality-assured product, and also increased knowledge (and perhaps an increased base of modules) that can be used for other projects. Credit for graphics: Romaine, Raúl Ruano Ruiz, Everado Coelho, Magnus Manske, Halfak, NIAID, all obtained from Wikimedia Commons under a CC4.0 or CC0 licence.

This approach is depicted in Figure 1.

## 3. To What Extent Have Past Successes Followed the Engineering Approach?

To answer this question, it is first necessary to define ‘success’. As every engineer knows, the success of a project can be measured in many different ways. The simplest, ‘does the device work?’, is one of the least stringent measures of success, while ‘is the device actually useful in the external world?’ is a much more stringent test, and one that is of much more interest to investors. A vast number of devices, in all fields of engineering, pass the first of these tests but fail the second, usually because some other thing works better (does more/is more economical/safer etc.). A great deal of public spending on synthetic biology has been justified in terms of real-world impact [20,21,22] and reviews of the field also stress its potential to find industrial, medical and agricultural application [23,24,25,26]. It is therefore reasonable to define, ‘success’ as a project that has resulted in a product that is either achieving practical industrial or medical use in the world outside the research laboratory, or that is at a stage of translation that makes such a use likely. This definition has a second advantage: where a field has an ideological attachment to a particular way or working (e.g., the engineering approach discussed here), it is obvious that the total work done in the field, whether useful or not, would be dominated by that way of working. Restricting our focus to projects that have found real-world success will break the circularity of this, and will allow us to examine whether that way of working has been a path to success. It should be noted that the definition of success used here excludes systems designed primarily for education and training, because the features of an education tool are often very different from that of a commercial product (contrast a typical ‘learn electronics by building a radio’ kit from a commercial radio receiver): education will be considered in Section 5 of this article.

Commercial development of synthetic biology is tracked by the *synbioproject* database, run independently by the Woodrow Wilson International Center for Scholars and funded by the Alfred P. Sloan Foundation [27]. This was used as an initial method for identifying projects finding real-world application. As backup strategies, Pubmed was searched with <“synthetic biology” commercial> and the general internet search engine DuckDuckGo was searched with the same term, and with ‘market’ in place of ‘commercial’, and the first 100 hits were examined. These two searches, though they returned much material, identified only one commercial or near-commercial application that was not already identified by the *synbioproject* database. Table 1 shows a variety of synthetic biological projects, which are already in the commercial market or that seem close to being, taken from this database and the literature reviews: the table excludes products in the database that are simply chemical derivatives products of other synbio projects, to avoid double-counting, and it also excludes what seem to be natural enzymes or trivial modifications of natural enzymes. It also excludes the many projects claimed in research papers to be of ‘potential’ commercial interest without any evidence of genuine commercial development. The projects in the table represent the main types of commercial or near-market applications of the present time. It is striking that the table is dominated by enzyme engineering and metabolic engineering, presumably because these are the applications of synthetic biology that can connect most easily with existing industrial processes and needs. To be clear, this type has been viewed as genuine synthetic biology in reviews of synthetic biology in *Nature* [28], *Science* [29], *Cell* [19] and *EMBO Reports* [15], and in reports from academic societies [10] and government agencies [20]: this current article follows that established practice. Examination of the publications describing the projects (references are in the table) reveals the extent to which the project used the engineering approach outlined in Section 2. None of them followed it entirely, and most did not follow even one of its elements. The one that did—the arsenic sensor—made some use of standard components but did not show the other features.

**Figure 2:**
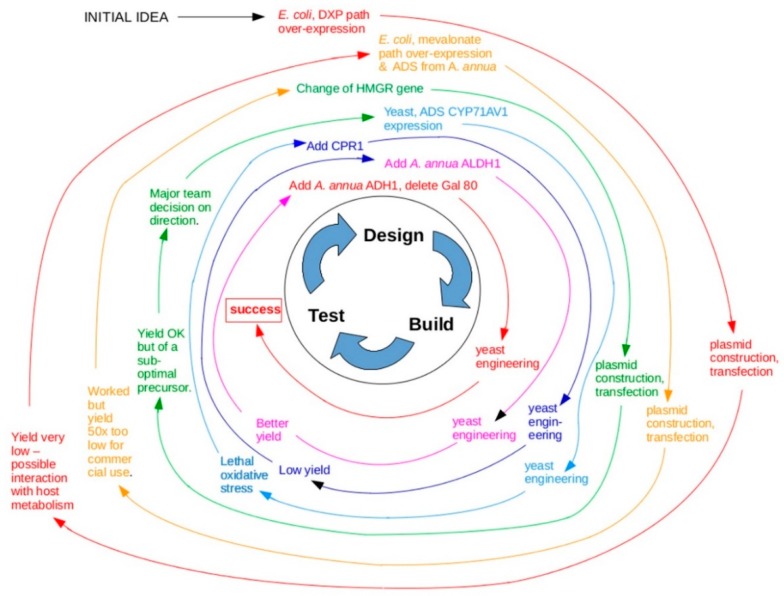
A representation of the progress of the project to produce artemisinic acid (precursor for the antimalarial compound artemisinin) by synthetic biological means. It should be noted that this diagram shows only the path to ‘first success’ and not to final commercial optimization, and also that it has been simplified in the interests of clarity: in reality there are more turns of this spiral, for example for strain choice, and some events (e.g., ADH, Gal80) have been presented together when in reality they were sequential and would have added another turn to the diagram. The story, which is even more complicated than this already messy diagram suggests, is told with great clarity in ref [32].

A good example of the gulf between the engineering approach that is claimed to be at the heart of synthetic biology, and the actual paths to success, is given by one of the most famous real-world applications, the synthesis of precursors of the anti-malarial drug, artemisinin (second entry, Table 1). A synthetic biology route to this was first described by Ro et al. [31], and worked by introducing enzymes from other organisms into a laboratory microorganism to create a new metabolic pathway, together with some inhibition of natural paths. According to the description in their paper, Ro et al. designed their pathway in a step-wise process, adding features (e.g., gene encoding enzymes), measuring the effect, and then adding the next feature; this was typically done with choices, the best-performing strain being chosen post-hoc rather than predicted quantitatively. There is no mention in the methods section of any predictive modelling. The genes used for the pathway enzymes were not drawn from any Registry or Catalogue of standard components, but were rather taken from other organisms with desired metabolic features and connected in a manner designed specifically for this one project. The performance of the system was tested according to assays designed for that specific system, and not according to any general scheme of metrics. The productivity of the system described in 2006 was inadequate for commercial production, and further work was needed to optimise the system [41]. Much of this optimisation was iterative, observations made on the results of early attempts (e.g., cells suffering oxidative stress) being used to inform later stages of the work, for example changing the transcription rate of some genes, and adding others [32]. The group responsible achieved something very important and laudable, but their path (summarised in Figure 2) was quite different from the engineering approach claimed by others to be at the heart of the field.

Similarly, synthetic biological production of D-Phenylglycine, a building block for the antibiotic cephalexin, was done by designing a metabolic pathway using components borrowed from other organisms. This pathway has been assembled, tested empirically, then improved in a stepwise manner by suppressing pathways that produced unwanted byproducts [36]. The final function of the system was tested using assays specific to the project rather than any generic suite of metrics. This pattern of empirical exploration in place of prediction is not confined to older projects or to those that involve pathway engineering. A recent synthetic biological project to confer additional interaction domains control on the CRISPR DNA-editing enzyme Cas9, for example, achieved its aim not through optimised, predictive design but through a carefully planned search strategy that tried almost every possible position of a control insert in a host protein to find the optimum [42]. These data were later built on to make hormone-controllable Cas9 and cpf1 but, even though these designs were based on the knowledge obtained from [42], it was still necessary for another stage of empirical testing of different versions of the design, only some of which showed satisfactory performance [43].

**Figure 3:**
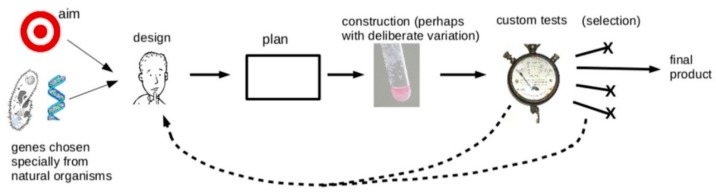
A diagrammatic representation of the general approach actually taken in the majority of the projects in Table 1. They used custom parts chosen specifically for the project, and achieved most optimization steps by testing different versions of the real wet-ware construct (not a computer model) and using the information to produce a better design. Many projects involved random variation and selection of the best alternative, rather than relying on purely predictive design. Credit for graphics: Romaine, Raúl Ruano Ruiz, Everado Coelho, Magnus Manske, Halfak, all obtained from Wikimedia Commons under a CC4.0 or CC0 licence.

So, we are forced to conclude that the actual application of the engineering approach to synthetic biology projects that are old enough to be in or near the market is not universal and may in fact be rare, and that typical projects proceed by processes much closer to those in Figure 3 than Figure 1. This conclusion is supported by the analyses of others [1,28,44]. In the words of a recent report from Cambridge Consultants, “The field of synthetic biology is still to become the fully- edged engineering discipline it is aiming towards” [45].

## 4. Should Synthetic Biology Follow the Engineering Approach in the Future?

The examples described in Section 3 show that synthetic biology projects can be successful even when operating in ways that involve a great deal of trial and error, with very little standardisation. Many authors have argued for replacement of this way of working with the engineering approach outlined in Section 2 [1,11,12,13,44,46,47]. Is this really desirable? This section will consider the advantages and disadvantages of each of the five features of that approach.

### 4.1. A Design Phase That Uses Predictive Models

Of all of the features of the engineering approach, this is the easiest to champion because predictive design would be very useful if it were one day to become possible. Building synthetic biological constructs in DNA, transferring them into cells, selecting for stable clones and verifying their behaviour is expensive in time and money: it would be much better to use models to optimise designs before they are realized in wet-ware. The power of models in conventional engineering stems from their predictive power, and that in turn stems from the precision with which the properties of construction components, and their ways of interacting, are known. This includes both the properties of active components (e.g., transistors) and also the confidently known inertness of supporting structures such as a radio chassis or printed circuit board. Engineering components (transistors, gears, chassis) are themselves the product of design and they are therefore very well understood. The components of synthetic biology, which are generally derived and adapted from natural molecules and gene sequences, are not understood nearly as well (even when the genes are synthesized de novo, the elements of proteins for which they code are usually derived from the study of functional domains in natural proteins). More critically, the properties of host cells are understood only in outline: the whole science of cell and molecular biology exists precisely to work these details out, and there are no signs that the enterprise is close to completion. In the light of this, the arrogant way in which some synthetic biologists dismiss the host cells as a mere ‘chassis’ is somewhat surprising.

The fact that we cannot yet model the behaviour of synthetic systems with precision, and that we may not be able to do so in the foreseeable future, does not mean that modelling has no place in the design phase. It can certainly be used to explore ideas with idealised components, and it can be very useful for sensitivity analysis (determining which parameters are critical to a system, and which can be anywhere in a wide range without disrupting function). Its power might increase if systems were to be measured in standard ways and the results fed back into models, either explicitly or through machine learning techniques. However, in the absence of complete data on components and host biology, modelling cannot be relied upon to find the optimum design, so a degree of parameter space-searching in the wet-ware phase will remain necessary for projects for which efficiency is critical.

### 4.2. Designing as Much as Possible Using Standard, Well-Characterized Components

From the dawn of 21st century synthetic biology, leading authors have argued for the construction of libraries of standard, well-characterised parts from which modules and devices can be assembled, in much the same way that radios, televisions and karaoke machines are assembled from standard electronic components (reviewed in [48]). To anyone with a sense of engineering history, the wisdom of this may appear self-evident. Joseph Whitworth’s standardisation of machine screw threads, making nuts of a given specification interchangeable so that they did not have to be custom-made to fit a particular machine screw or bolt, made manufacture and maintenance of machinery much faster and more economical [49]. The development of standard electronic components in place of the hand-built resistors and hand-blown vacuum tubes of the early days made the radio, television and information ages possible. Similar stories can be told in almost all other fields of engineering. This, together with educational considerations, prompted Tom Knight to found the Registry of Standard Biological Parts (‘Biobricks’: [50,51]), a set of generic elements (promoters, reporters, terminators etc.) that were classified, schematised, listed and described in much the same way that components appear in an electronics catalogue. The Registry is open to all and can be examined at https://parts.igem.org/Main_Page.

The use of standard parts confers three advantages in conventional engineering; (i) it simplifies design because the designer can specify the use of a component with known, well-described properties and need not normally worry about its internal details of that component or how it is manufactured; (ii) it brings economy of manufacture, because the same basic component can be used as a standard part in a large number of different final devices so some manufacturers can devote themselves to manufacturing components at high volume; (iii) maintenance is made much easier by the fungibility of components carrying the same part number. In synthetic biology, the value of the first advantage depends on how known the properties are, a problem that has already been discussed in Section 4.1. The last two advantages are irrelevant to synthetic biology; living systems, once made, reproduce themselves so that almost all cost of manufacture is the production of the prototype only, and cells maintain themselves.

There are, on the other hand, two potential disadvantages to using standard parts. The first is inefficiency; in most cases, the combination of standard parts required to perform a task will be larger and more complex than a custom part designed for the job. This is especially true in fields like metabolism, for which a route-by-standard-parts may be much longer than one mediated by a custom enzyme (or one borrowed from another organism that happens to feature a desired transition in its own metabolism). Anyone involved in designing or maintaining mechanical or electronic devices will be familiar with the compromises accepted in the interests of avoiding the expense of an optimised, custom component. This is not important in typical consumer or industrial applications, where there is no great penalty for using a few more components or a little more energy than strictly necessary. In living cells, however, any additional metabolic load can be deleterious and create selection pressures favouring cells that manage to inactivate the synthetic construct. The other, long-term disadvantage of standarization second is vulnerability. One of the strongest defences of natural organisms against microbial attack is their diversity, maintained by gene shuffling through sexual reproduction. Clones of identical organisms grown in monoculture are very vulnerable to any pathogen adapted to them, as demonstrated by the Irish potato blight and its associated famine [52]. Economies that rely on standardized interconnections of standardized computers are now routinely attacked by criminals intent on disrupting their activities or holding them to ransom. The standardisation of systems and interconnection protocols makes these attacks much easier since so many devices work in the same way. If our agricultural, energy and industrial economies come to rely on synthetic biological devices, we may be much safer if they have been built from diverse and unique custom parts rather from a common set of standard components.

### 4.3. Hierarchical Design Using Previously-Characterised Modules

The use of modules (collections of components arranged for a particular task) is very common in the engineering industry; motors, gearboxes, amplifiers, memory boards and prefabricated roof trusses are familiar physical examples. Ubiquitous, but less obvious except to software engineers, is the extensive use of libraries of pre-written modules that are used in most everyday computer applications; these libraries can be called upon in code and are included when the software is compiled (translated, by existing software, from human-friendly languages to machine code).

There are two main advantages of building a complex project from modules. The first and most obvious is that it allows the use of pre-existing modules to perform some functions, instead of having to design designing everything from first principles. The cliché about ‘not re-inventing the wheel’ is precisely about using an existing hub-and-spoke-and-tyre module because it is available. If a module performs the exact task required, and nothing that is not required, then reusing it in a new design may make sense. It is common, however, for modules to have features additional to the minimum required and therefore to be larger than they need to be. The effect of using them anyway can easily be appreciated by the rapid rise in the size and memory requirements of computer applications, a trend captured in the informal term ‘bloatware’. In two illuminating essays, the programmers Nikita Propokov and Jordan Scales examine many examples of this [53,54]. A typical one is the current Google Keyboard App, the function of which is to draw 30 ‘pressable’ keys on a tablet screen: this app uses three times more memory than the entire Windows 95 operating system did! They also discuss how software has become so terribly inefficient. Two strong themes are the use of modules with unnecessary functionality in addition to the function needed, and the creation of new functions by building on layers of existing modules, which were themselves built on deeper layers, creating webs of interactions and dependencies that increase the size of applications, decrease their speed, and give them far more unpredicted behaviour than software written from scratch would have.

The effects of this in computers are almost tolerable because computer hardware has become more powerful at a rate that has kept up with decreasing efficiency of software (routine tasks on an office computer, such as scrolling through a document or finding an e-mail, take about as long now as they did 20 years ago; the bloatware adds new, less commonly used features). However, the capacity of living cells to provide resources for additional systems is limited and will not rise in the way that the capacity of computer systems has. We cannot make the same mistakes in designing synthetic biology systems that we have made in writing consumer and business software, because host cells will simply not support the resulting mess. Instead, synthetic biological devices will need to be as optimized as possible, with no use of modules with unnecessary features.

There is a second advantage to modular construction that applies even where no pre-existing modules exist. In conventional engineering, modular construction allows abstraction and hierarchical design [13,55], allowing a complex project to be broken down into sub-units that can be designed independently: as long as the connections between the modules (power supply, signal level and types etc.) are well-defined, design teams of one module can ignore the internal architecture of others. This ‘decoupling’ [13] works because the interactions of modules can be limited, for example to only those points where wires connect one module to another. Sometimes, even in conventional engineering, problems arise when interactions are not as controlled as expected (radio-frequency emissions, heat and vibration being common media of unexpected coupling). In synthetic biology, most components diffuse freely in the cell and their interactions are not limited by wires. In electronics, teams making different electronic modules are free to use exactly the same bistable memory latch device: memory latches exist in synthetic biology too, and most use diffusible proteins to control transcription (reviewed in [56]) so that, if two modules used the same latch design internally they would interact strongly. Design teams of one module therefore need to be very aware of the architecture of other modules so that they do not use components that interact. The problem cannot be solved merely by avoiding use of identical components; potential interactions can be subtle and hard to predict in advance, especially when they proceed via the host physiology [57]. This does not mean that modular construction cannot or should not be used, but it does mean that the gains from this way of thinking will not be as great as they are in conventional engineering, and that they have higher risks of introducing unintended ‘bugs’ into the system.

### 4.4. Manufacture Using Reliable, Quality-Assured Systems

This is relevant, and largely achieved, for manufacture of the lowest-level components such as DNA sequences, and for the use of established synthetic biological systems for making drugs safe enough for human use. It is much less relevant for the production of the engineered cells themselves, because it is usually so simple to select the one-in-10,000 cells that have been made properly and use the self-reproducing feature of life to increase their numbers as required.

### 4.5. Testing of Devices Using Standardised Measurement Techniques

The creators of a synthetic biological device intended to perform a specific task will almost certainly have to apply unique tests to it, designed around the desired outcome, to verify its performance. They may also choose to apply standard tests to the whole system and to its subcomponents [58]. The value of performing standard tests is more to the community as a whole than to the project itself [46]. By associating devices and cellular and environmental contexts with standard measures of performance, and submitting this data to an open repository, synthetic biologists contribute to the development of better predictive models for the design phase. The utility of this will be greatest when the components themselves are drawn from a standard set, and least when they are custom-built for only one purpose. There is therefore a kind of cultural feedback in operation: if a team commits to use of standard components it will make sense for them to commit to standard measurements as well (in addition to any custom ones), for the sake of the whole standards-centred community. If a team eschews standard components for custom design, there is much less point in their using standard measurements. There is scope, however, for something like the ‘yellow card’ scheme for reporting adverse drug interactions or side-effects, run by the UK’s Medicines and Healthcare Regulatory Agency (MHRA). If synthetic biologists using even very non-standard components could submit reports of any unexpected interactions (between components and between components and host cell) in a simple way, more could be learned about risk of failure, if only at the level of identifying, statistically, host cell systems most likely to be involved in unexpected interactions.

## 5. Where the Engineering Metaphor Is Useful

Because this article is written as a challenge to a vigorously promulgated but, in the author’s view, unhelpful dogma that synthetic biology always uses, or should aspire always to use, an engineering approach, there is a risk that it may read as unbalanced. To be clear, I am not arguing that there is no place in synthetic biology for practices derived from conventional engineering—only that these must not be allowed to confine the development of the field. There are clearly aspects of synthetic biology in which the engineering metaphor is appropriate.

The first and most obvious aspect is in education. The iGEM series of annual competitive workshops (reviewed in [18,59]), at which many young people gain their first taste of synthetic biology, depends for its success on the BioBricks Registry of Standard Biological Parts [60,61]. These adhere to a well-defined set of standards to ensure a reasonably high chance of their being assembled easily in a large variety of combinations [51]. It is precisely because student teams can choose well-characterized parts ‘off the shelf’, as they might chose components for a project in electronics, mechanics or hydraulics, that it has been possible for inexperienced teams to achieve so much in so short a time. Use of standard parts is of course much quicker than designing or modifying custom ones. However, it must be remembered that the primary aims of iGEM are engagement and education, not the design and production of an optimized system for real-world use. For education, speed of design and assembly are important and final efficiency is not; the effectiveness of the process as a learning experience matters much more than the effectiveness of the cells doing their new job. Some iGEM-derived devices are taken on towards real-world applications (e.g., the arsenic sensor of [62]), but this process usually requires optimization steps [39,63,64] that move away from the standard components and involve careful custom-engineering and evaluation of several different alternatives whose properties could not be predicted (or at least were or in fact predicted) in advance.

The most important use for the engineering-derived practice of standardisation is probably in the area of metrics and description. Many areas of science have been improved by the adoption of codes of practice that specify a minimum data set to describe a particular analysis. The MIAME standard for description of micro-array experiments and the MISFISHE standard for description of in-situ hybridisation experiments, and MIBBE for biology in general are three outstanding examples [65,66,67]. Adoption of minimum data sets for describing or characterising synthetic biological systems, many of which already exist [8], will be very useful in aiding comparison between performance of different devices, but it is again important that the standards are flexible enough to be able to be adapted to new types of devices or measurements that were not thought of when the standards were drawn up. It would be a tragedy if an insistence on rigid adherence to a standard system of metrics and description were allowed to prevent the development of new devices for which the old standards are not appropriate. A simple method of dealing with this, a method that is drawn from open-source software engineering, is ‘forking’: making a copy of an established open system (of standards, in this case), without the need for any permission from the original developers of that system, and then altering it for a particular new purpose and making that altered version fully available for all to use [68].

## 6. Conclusions

This article has argued that the dogma that synthetic biology already operates like, or that it must in the future operate like, conventional engineering is false. That does not mean that the engineering approach should be excluded; merely that it should not be insisted upon. The unique features of biological systems, in particular that we still understand them so completely, makes the classical engineering approach of less value than it is in the world of non-living technologies and makes other approaches more valuable than they would be in the physical world.

There is a deeper point to be made. The authors cited at the beginning of this article, and many others like them, wish to improve biological technology through the application of knowledge and practices adopted from conventional engineering. What might be more fruitful is a flow of ideas in the opposite direction. Biologists, including their main ‘engineering’ flank, surgeons, physicians, and veterinarians, have evolved ways of working that allow them to manipulate living systems to achieve desirable outcomes by adapting their approach to the characteristics of life, rather than the other way around. When a natural system is not understood in detail (which is almost always the case), biologists start by optimising not the design of their new device (e.g., a drug), but by optimising the design of a high-throughput screen that will explore a vast volume of parameter space (chemical structure, concentration, timing, etc.) to find the candidate that works best [69,70,71]. When they wish to modify a cell line or organism, either by deliberate genetic manipulation or by random mutagenesis and breeding, they design selection pressures to mimic evolution, so that even a very rare desirable genotype will come to dominate a culture within a few generations. They also often perform this iteratively, combining elements of promising lines randomly and again selecting for the best (this is the basis of classical breeding). None of this involves truly predictive modelling and the structure of the best device, when analysed retrospectively, is often a surprise.

This way of working is anathema to most classical engineers, but it has served our species well for thousands of years—far longer than industrial engineering—and even now crop breeding can still outperform genetic manipulation [72]. One field of engineering has been receptive to borrowing ideas from biology: at least in fields devoted to designing adaptive or ‘intelligent’ systems, genetic algorithms and neural net strategies, both based entirely on biological principles, have been adopted and are proving successful [73]. Of all the engineering disciplines, software is the most like biology anyway because it shares the attributes of rapid, almost cost-free reproduction and of very large numbers of variants being able to co-exist and compete in an ‘ecosystem’. However, given that one use of software is to conduct the predictive modelling at the beginning of the classical engineering process, it would be perfectly possible for this evolutionary, exploratory way of working to be used to generate and evaluate many models of a desired object (a submarine hull, say) and to find an optimum plan, even if the engineers do not understand explicitly why it is the optimum. The idea has been used to design optimum wiring networks [74] and the ‘evolved design’ of NASA’s ST5 spacecraft antenna is an example of a plan that arose this way [75]. The use of a ‘biological’ rather than conventional method of working on this project, a micro-satellite in which efficiency was very important, is telling, and very relevant to the restraints of synthetic biology.

So, in conclusion, synthetic biology has not always used the classical engineering approach so far, and there is no good reason that this approach should define it in the future. Rather than allowing classical engineering to constrain synthetic biology, we would be much wiser to borrow ideas from classical engineering when they can help us, and also to allow biologists’ ways of working to expand and enrich the realm of engineering. With the right kind of synthesis, the title of this paper will become meaningless, as ‘engineering’ will have expanded to embrace a much richer variety of ways of working, their unifying feature being the way that they result in the optimum solution to any given problem.

## Figures and Tables

**Table 1:** Attributes of a selection of successful, real-world synthetic biological projects. The use of components so standard across biology that they are not particularly associated with synthetic biology, such as commercial plasmids and cloning systems, has been ignored in the ‘standard components’ column: this column refers instead to components drawn from a registry of standard parts intended for synthetic biology.

Project	How	Ref	Status	Predictive Models?	Std Components?	Modular?	Standard Testing?
Accelerase Trio (for biomass conversion)	Enzyme engineering	[30]	Commercial	Unknown	No (started with natural enzymes chosen for this specific purpose)	No	No (custom)
Arteminisic acid synthesis (drug)	Metabolic path engineering	[31,32,33]	Commercial	No (design was optimized empirically)	No (custom, selected from other organisms)	No	No (custom)
Bioisoprene synthesis (for rubber)	Metabolic path engineering	[34]	Near-market	No (empirical testing of alternatives was used instead)	No (custom, selected from other organisms)	No	No (custom)
Cellic Ctec (enzyme for biomass conversion)	Enzyme engineering	[35]	Commercial	Unknown	Enzyme engineering	No	No (custom)
Cephalexin synthesis (drug)	Metabolic path engineering	[36]	Commercial	No (design was optimized empirically)	No (custom, selected from other organisms)	No	No (custom)
Biodiesel	Metabolic path engineering	[37]	Near-market	No (a model was used to inform overall plan and to assist with analysis but not to predict based on components)	No (custom, selected from other organisms)	No	No (custom)
Fuelzyme amylase	Enzyme engineering	[27]	Commerical	Unknown	No (custom, selected from other organisms)	No	No (custom)
Luminase PB-100	Enzyme engineering	[27]	Commercial	Unknown	No (custom)	No	No (custom)
Resveratrol synthesis (drug)	Metabolic path engineering	[38]	Near-market	No (design was optimized empirically)	No (custom, selected from other organisms)	No	No (custom)
Arsenic sensing	Novel genetic ‘circuit’ with sensor	[39,40]	Near-market	Yes (ODE-based model using estimated parameters for performance prediction and sensitivity analysis)	Yes (BioBricks)	No	Partially
